# *QuickStats*: Percentage of U.S. Women Aged 21–65 Years Who Never Had a Papanicolaou Test (Pap Test),* by Place of Birth and Length of Residence in the United States^†^ — National Health Interview Survey, 2013 and 2015^§^

**DOI:** 10.15585/mmwr.mm6612a9

**Published:** 2017-03-31

**Figure:**
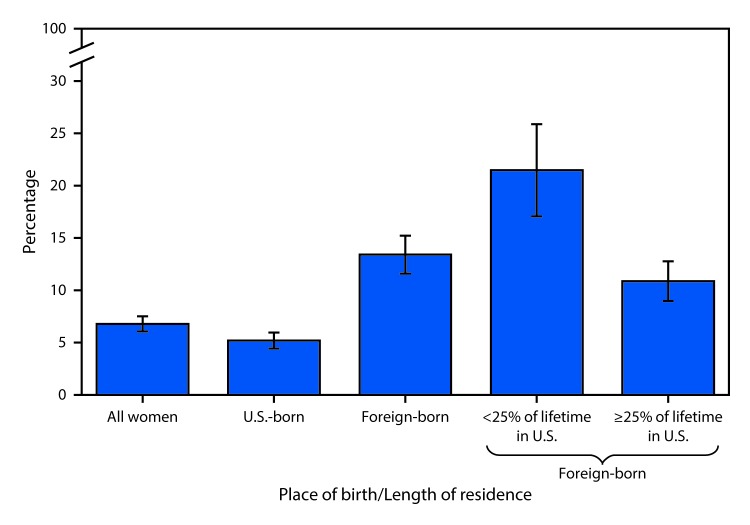
In 2013 and 2015 combined, 6.8% of U.S. women aged 21–65 years had never received a Pap test in their lifetime. Foreign-born women were more than twice as likely as U.S. born women to have never received a Pap test (13.4% versus 5.2%). Foreign-born women who lived in the United States for <25% of their lifetime were almost twice as likely as those who resided in the United States for ≥25% of their lifetime (21.5% versus 10.9%) to have never received a Pap test.

**Source:** National Health Interview Survey, 2013 and 2015 combined. https://www.cdc.gov/nchs/nhis.htm.

